# Retraction: A fine balance of dietary lipids improves pathology of a murine model of VCP-associated multisystem proteinopathy

**DOI:** 10.1371/journal.pone.0349181

**Published:** 2026-05-12

**Authors:** The PLOS One Editors

After this article [1] was published, concerns were raised regarding results presented in Fig 1 and the reporting of the animal experiments.

Specifically:

- Part of the Fig 1B VCP^R155H/+^ ND panel appears similar to part of the Fig 1D VCP^R155H/+^ 9% LED (7 months+) panel.
- The Materials and Methods section does not report humane endpoints or the method of euthanasia used in the animal experiments.

Author VEK stated that an error occurred during preparation of Fig 1. They provided updated panels for Figs 1B–1D, and some of the original underlying image data for these figures. Upon editorial review, PLOS identified additional concerns for the updated panels and underlying images provided that bring into question the reliability of the reported results.

During post-publication discussions, author VEK provided quantitative data underlying Figs 1F, 4E, 5B, and 5C. Editorial assessment of these underlying quantitative data raised concerns for the reliability of the error bars presented in these figures in [1].

Regarding the animal experiments, author VEK stated that the animals were treated humanely according to the IACUC protocols, the animals were either found dead in their mother’s cages or were sacrificed for the study, and the method of euthanasia used was CO_2_ inhalation followed by cervical dislocation. They further stated that during the survival studies, the animals’ health and behavior were monitored daily to see if the homozygote animals were alive, and provided the University of California-Irvine IACUC protocol #2007-2716-2. This document showed animal wellbeing was assessed by monitoring body temperature, weight and behavior, and that animals were euthanized if they showed weight loss, behavioral problems, or sudden illness.

In light of the above unresolved image and data concerns that question the reliability and integrity of the reported results, the *PLOS One* Editors retract this article [1].

LB, VEK, and AN did not agree with the retraction and stand by the article’s findings. VEK and AN apologize for the issues with the published article. KJL, NW, CN, and BT either did not respond directly or could not be reached.
